# The interaction between *TERT* promoter mutation and *MGMT* promoter methylation on overall survival of glioma patients: a meta-analysis

**DOI:** 10.1186/s12885-020-07364-5

**Published:** 2020-09-21

**Authors:** Huy Gia Vuong, Thu Quynh Nguyen, Tam N. M. Ngo, Hoang Cong Nguyen, Kar-Ming Fung, Ian F. Dunn

**Affiliations:** 1 Department of Pathology, Oklahoma University Health Sciences Center, Oklahoma City, OK 73104 USA; 2 Stephenson Cancer Center, Oklahoma University Health Sciences Center, Oklahoma City, OK 73104 USA; 3 Faculty of Medicine, Pham Ngoc Thach University of Medicine, Ho Chi Minh City, 700-000 Vietnam; 4 Department of Neurosurgery, Oklahoma University Health Sciences Center, Oklahoma City, OK 73104 USA

**Keywords:** Glioma, Glioblastoma, TERT, MGMT, Temozolomide, Overall survival, Meta-analysis

## Abstract

**Background:**

There are controversial results concerning the prognostic implication of *TERT* promoter mutation in glioma patients concerning MGMT status. In this meta-analysis, we investigated whether there are any interactions of these two genetic markers on the overall survival (OS) of glioma patients.

**Methods:**

Electronic databases including PubMed and Web of Science were searched for relevant studies. Hazard ratio (HR) and its 95% confidence interval (CI) for OS adjusted for selected covariates were calculated from the individual patient data (IPD), Kaplan-Meier curve (KMC), or directly obtained from the included studies.

**Results:**

A total of nine studies comprising 2819 glioma patients were included for meta-analysis. Our results showed that *TERT* promoter mutation was associated with a superior outcome in MGMT-methylated gliomas (HR = 0.73; 95% CI = 0.55–0.98; *p*-value = 0.04), whereas this mutation was associated with poorer survival in gliomas without MGMT methylation (HR = 1.86; 95% CI = 1.54–2.26; *p*-value < 0.001). *TERT*-mutated glioblastoma (GBM) patients with MGMT methylation benefited from temozolomide (TMZ) treatment (HR = 0.33; 95% CI = 0.23–0.47; *p*-value < 0.001). MGMT methylation was not related with any improvement in OS in *TERT*-wild type GBMs (HR = 0.80; 95% CI = 0.56–1.15; *p*-value = 0.23).

**Conclusions:**

The prognostic value of *TERT* promoter mutation may be modulated by MGMT methylation status. Not all MGMT-methylated GBM patients may benefit from TMZ; it is possible that only *TERT*-mutated GBM with MGMT methylation, in particular, may respond.

## Background

Gliomas are among the most common primary brain tumors in both adults and children [1]. Historically, glioma classifications and treatment options have been based on histological phenotypes, which lead to inconsistent outcomes. Recently, the 2016 revised classification of the World Health Organization (WHO) prioritized molecular signatures in pathologic determination. Brain tumors diagnosis, treatment, and prognosis were dependent on not only phenotypes but also genotypes [2–4]. This new classification emphasized the essential role of molecular testing in tailoring clinical decision and predicting patients’ survival, in which *IDH1* and 1p/19q status play an especially central role to classify the glioma tumors [1].

An emerging literature has provided an insight into the molecular characteristics of glioma which has enhanced the accuracy of diagnosis and prognosis. *Telomerase reverse transcriptase* (*TERT*) promoter mutation is one such marker. *TERT* plays an important role in telomerase activation leading to the immortality of malignant cells [5]. *TERT* C228T and C250T were the most common mutations [5]. Mutation of *TERT* promoter as a genetic event is frequently detected in 60–75% of glioblastomas (GBM), and associated with a poor prognosis [5, 6]. While *TERT* promoter mutation showed a poor survival prognosis in glioma patients, *O*^*6*^*-methylguanine-DNA methyltransferase* (MGMT) methylation has long been recognized as an important factor in treatment decisions [7], and is also a positive prognostic factor [8–12]. Our previous study, along with others, indicated that the prognostic value of *TERT* promoter mutation in gliomas is influenced by the status of *IDH* mutations [5, 13–15].

The prognostic inter-relationship between *TERT* promoter mutations and MGMT methylation status has been unclear. The combination of *TERT* promoter mutations and MGMT promoter methylation has defined subgroups with noticeable responses to current treatments [10]. Some data have suggested that glioblastoma patients harboring MGMT methylation have a different prognosis depending on *TERT* promoter mutation status [16]; on the other hand, some studies have reported no association in the co-occurrence of *TERT* promoter mutation and MGMT methylation in glioma patients [14, 17–19].

In this study, we conducted a comprehensive meta-analysis to further understand whether *TERT* promoter mutation has any interaction with MGMT promoter methylation on overall survival (OS) of glioma patients.

## Methods

### Literature search

Our search was limited in two electronic databases including PubMed and Web of Science, from inception to October 2019. The below search terms were used: TERT AND MGMT. Potential studies were also searched by reviewing the citations within the included studies and reviews. We followed the recommendations of Preferred Reporting Items for Systematic Review and Meta-analysis (PRISMA) statement [20] (Supplementary Table 1).

### Selection criteria and abstract screening

We brought all searched results from two electronic databases above into EndNote (Thomson Reuters, PA, US). Duplicated research papers were discarded. Titles and abstracts were independently assessed by two reviewers. We included research papers providing data regarding prognosis of MGMT promoter methylation and *TERT* promoter mutation on glioma patients’ overall survival (OS). We excluded studies if they were studies on brain tumors other than glioma; studies lacking data on MGMT promoter methylation or *TERT* promoter mutation; case reports; reviews; posters, conference papers, theses or books; and duplicated articles. Any differences in opinions between reviewers were resolved by discussion and consensus.

### Full-text screening and data extraction

Two reviewers independently reviewed all relevant research papers’ full text. Potential data were extracted into a designated worksheet. The following data were extracted from full texts: authors, institution, city, country, year of publication, study design, number of patients, demographics (age and gender), WHO grade, follow-up periods, data of hazard ratio (HR) and its 95% confidence intervals (CIs) on OS, and adjusted covariates if available. We directly obtained HR and its 95% CI information from full text papers or calculated from the provided individual patient data (IPD). If not applicable, data were indirectly calculated from KMC using the methods by Tierney et al [21]. Any disagreements between two reviewers, if present, were solved again by discussion and consensus. Besides, we tried to contact the authors via email to request additional data or IPD if data were insufficiently provided in the original papers.

### Quality assessment and risk of bias analysis

We evaluated the quality of included studies in our meta-analysis using the Newcastle – Ottawa Scale (NOS) [22]. Two reviewers independently scored the number of stars for cohort or case-control studies based on a developed checklist [22]. The maximum number of star (NOS) given is nine; studies awarded six stars or more were considered moderate to high-quality studies, and those with fewer than six stars were considered low-quality studies.

### Statistical analysis

We used the multivariable Cox regression model with backward stepwise, analyzed by R (http://www.R-project.org), to assess the effects of *TERT* promoter mutations and MGMT promoter methylation on OS. Proportionality assumptions of the Cox regression models were assessed by log-log survival curves and with the use of Schoenfeld residuals. Hazard ratios are presented as mean and 95% confidence intervals. HRs for OS were calculated from IPD, provided in original articles or via email request, and adjusted for confounding factors (age, gender, and WHO grade). When investigating the prognostic implication of *MGMT* promoter methylation in GBMs, data regarding chemotherapy (TMZ) was added into the adjusted covariates. Because of limited data, we did not include other molecular biomarkers such as *IDH* mutation or 1p/19q co-deletion as adjusted factors.

Pooled HRs for OS were calculated using the random-model effect weighted by the inverse variance method. An HR > 1 indicated a worse prognosis in glioma patients with genetic alterations. If the authors provided several HR numbers in the same study, we selected the most powerful one for primary outcome analysis in ideal order: adjusted HR > unadjusted HR > HR estimated from KMC. We used Review Manager 5.3 program (Cochrane Collaborative, Oxford, UK) for our analysis.

We assessed among-study heterogeneity using *I*^*2*^ statistic which explored included studies’ total variation is not by chance [23]. An *I*^*2*^ statistic of 25–50% showed a low amount of heterogeneity, and > 50% indicated a high amount of heterogeneity [24]. The sources of heterogeneity were examined by using (i) subgroup analysis and (ii) sensitivity analysis.

### Risk of bias assessment

Egger’s regression test and funnel plot were done for evaluating the presence of publication. A *p*-value of less than 0.05 was considered statistically significant publication bias.

## Results

**Fig. 1:**
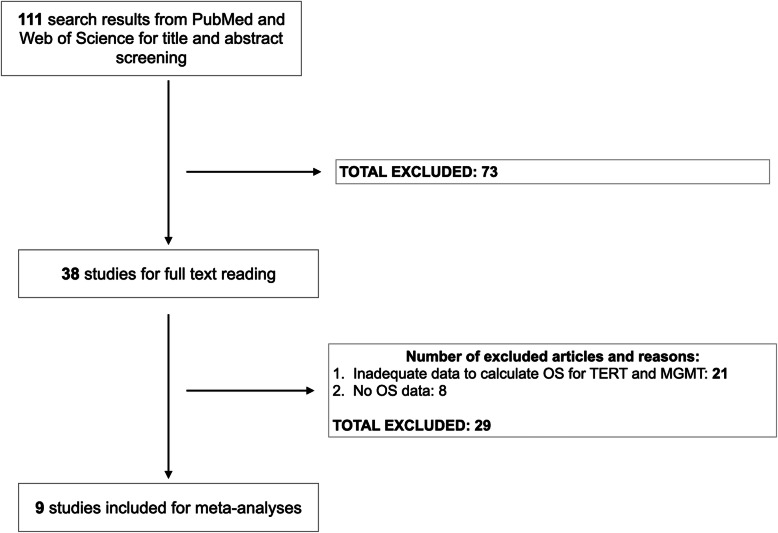
Study flowchart. Abbreviations: OS, overall survival

We found 111 articles for abstract screening in which 38 studies were included for full text reading. After the full text screening step, we included eight papers satisfying our selection criteria. After contacting the corresponding authors of selected studies for potential unpublished data, we received a response from one paper providing their IPD [25]. Finally, a total of nine studies were included for meta-analyses comprising of 2819 glioma patients (Fig. 1) [16, 25–32]. The baseline characteristics of these studies were presented in Table 1.

**Table 1:** Baseline characteristics of 9 included studies

Study	Institute	Country	No. of cases			NOS domain		
			LGG	GBM	Total cases	Selection	Comparability	Outcome
Arita 2016 [26]	Multicenter	Japan	421	337	758	4	0	3
Ceccarelli 2016 [27]	The Cancer Genome Atlas	USA	516	606	1122	4	0	3
Nguyen 2017 [16]	Multicenter	USA	0	303	303	4	0	3
Park 2014 [25]	Seoul National University Hospital	Korea	0	48	48	4	0	2
Picart 2018 [28]	Lyon University Hospital	France	0	17	17	4	0	2
Picca 2018 [29]	OncoNeuro Tek	France	30	86	116	4	0	2
Sasaki 2018 [30]	Multicenter	Japan	26	114	140	4	0	3
Weller 2015 [31]	Multicenter	Germany	137	0	137	4	0	3
Ye 2019 [32]	Xiangya Hospital	China	0	178	178	4	0	2

Abbreviations: *LGG* Lower-grade glioma, *GBM* Glioblastoma, *NOS* Newcastle Ottawa Scale

The NOS tool was used to assess the quality of each included study. The number of stars awarded to each of them ranged from six to seven stars. Details of given stars within each NOS domain were shown in Table 1.

### The clinical implication of *TERT* promoter mutation on OS in association with MGMT methylation status in gliomas

**Fig. 2:**
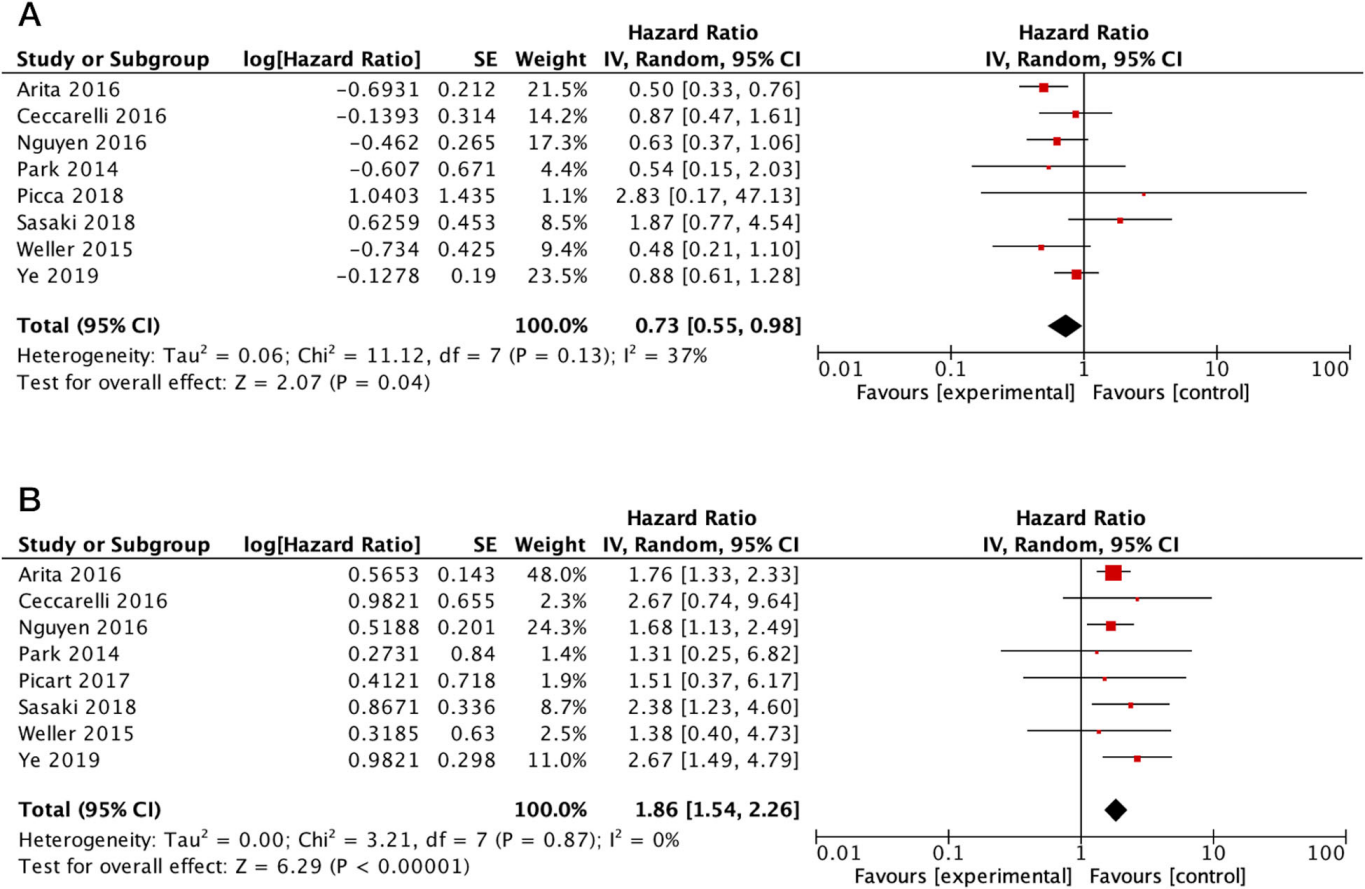
Forest plots illustrating the prognostic implication of *TERT* promoter mutation in MGMT-meth (**a**) and MGMT-unmeth (**b**) gliomas. Abbreviations: IV, inverse variance; CI, confidence interval; SE, standard error

In MGMT-methylated (MGMT-meth) gliomas, the presence of the *TERT* promoter mutation was associated with an improved OS (HR = 0.73; 95% CI = 0.55–0.98; *p-*value = 0.04). There was a low heterogeneity among the included studies (*I*^2^ = 37%) (Fig. 2a). After omitting the Sasaki et al. study [30], there was no change in the overall result and the among-study heterogeneity was insignificant (HR = 0.68; 95% CI = 0.54–0.85; *I*^2^ = 6%).

On the other hand, *TERT* promoter mutation was an indicator of worse outcome in MGMT-unmethylated (MGMT-unmeth) gliomas (HR = 1.86; 95% CI = 1.54–2.26; *p*-value < 0.001) (Fig. 2b). No heterogeneity was detected among the analyzed data (*I*^2^ = 0%).

### The prognostic impact MGMT promoter methylation stratified by *TERT* promoter mutation status in gliomas

Calculated data were adjusted for age, gender, and WHO grade, if applicable. MGMT promoter methylation was associated with a superior OS in both *TERT-*mut (HR = 0.29; 95% CI = 0.21–0.39; *I*^2^ = 44%) and *TERT-*wt gliomas (HR = 0.54; 95% CI = 0.39–0.74; *I*^2^ = 19%). Sensitivity analysis showed a robust result and the among-study heterogeneity was completely removed.

### Subgroup analyses regarding the impact of *TERT* promoter mutation and MGMT methylayion on overall survival of LGGs and GBMs

Table 2 shows that among MGMT-met LGGs and GBMs, *TERT* promoter mutation did not have a significant impact on OS (*p-*value = 0.18 and 0.11, respectively). On the other side, this mutation resulted in a compromised OS among MGMT-unmet LGGs and GBMs.

**Table 2:** Subgroup analyses concerning the impact of *TERT* promoter mutation and MGMT methylation on overall survival of LGGs and GBMs

Subgroups			HR	95% CI	*p*-value	*I*^*2*^*(%)*
LGG	*TERT*-mut vs *TERT*-wt	MGMT-met	0.62	0.31–1.24	0.180	60
		MGMT-unmet	1.47	1.01–2.16	0.045	0
GBM		MGMT-met	0.79	0.59–1.05	0.110	17
		MGMT-unmet	1.93	1.55–2.41	< 0.001	0
LGG	MGMT-met vs MGMT-unmet	*TERT*-mut	0.26	0.11–0.63	0.003	65
		*TERT*-wt	0.41	0.26–0.64	< 0.001	0
GBM		*TERT*-mut	0.31	0.25–0.39	< 0.001	0
		*TERT*-wt	0.85	0.67–1.07	0.160	0

Abbreviations: *CI* Confidence interval, *met* Methylated, *GBM* Glioblastoma, *HR* Hazard ratio, *LGG* Lower-grade glioma, *mut* Mutated, *unmet* Unmethylated, *wt* Wild-type

In *TERT*-mut and *TERT*-wt LGGs and GBMs subgroups, MGMT methylation was associated with a favorable OS in most of the subgroups. Heterogeneity was present among a few LGG subgroups.

### TMZ treatment in MGMT-methylated GBM patients

**Fig. 3:**
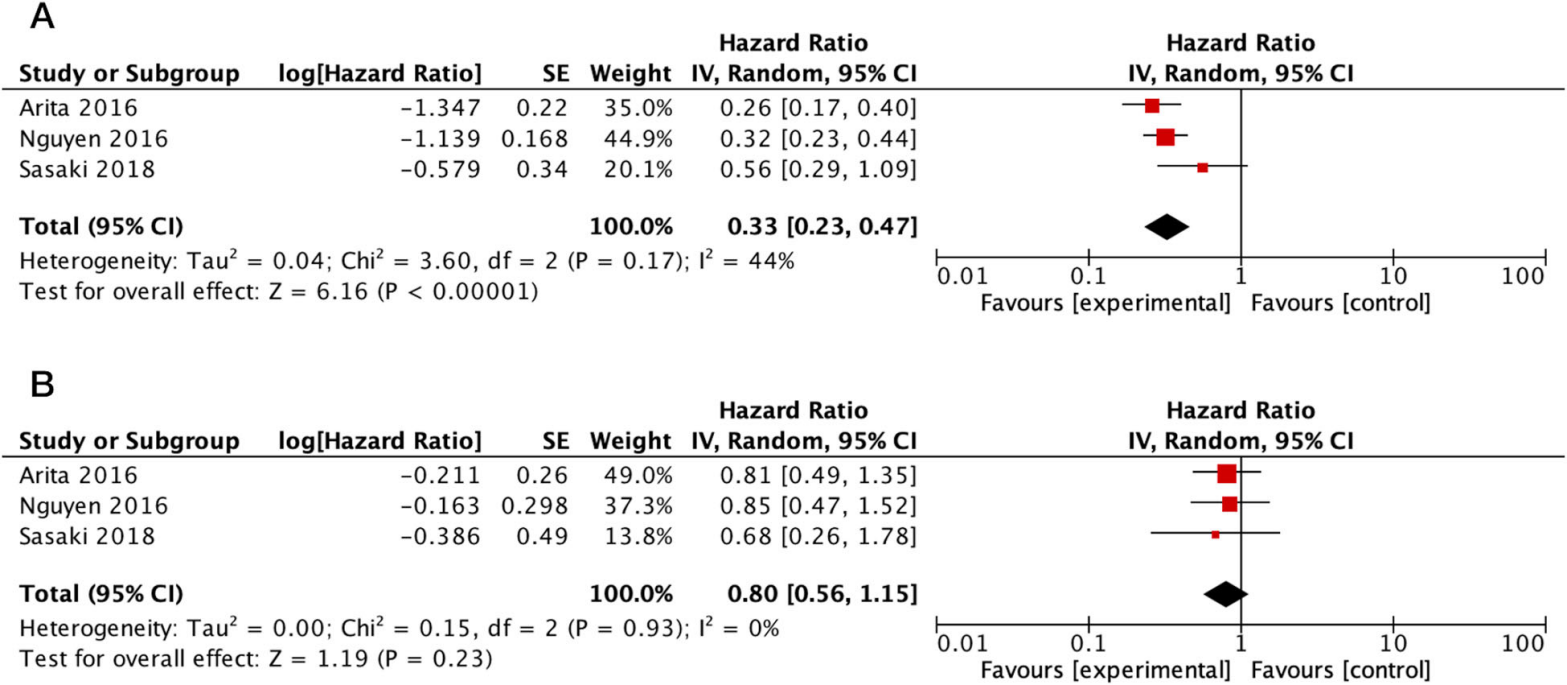
Forest plots illustrating the clinical significance of MGMT promoter methylation in *TERT-*mut (**a**) and *TERT-*wt GBMs (**b**) treated by TMZ. Abbreviations: IV, inverse variance; CI, confidence interval; SE, standard error

Three studies with sufficient data regarding chemotherapy treatment were included for meta-analysis [16, 26, 30]. While focusing on GBMs and adjusted for age, gender, and TMZ treatment, only *TERT*-mut GBM patients with MGMT methylation appeared to benefit from TMZ treatment (HR = 0.33; 95% CI = 0.23–0.47; *I*^2^ = 44%), whereas MGMT methylation did not appear to be associated with improvement in OS in *TERT-wt* GBMs (HR = 0.80; 95% CI = 0.56–1.15; *I*^2^ = 0%) (Fig. 3). After omitting data from the Sasaki et al. study [30], the among-study heterogeneity in the former analysis completely disappeared and the overall result was unchanged (HR = 0.30; 95% CI = 0.23–0.39; *I*^2^ = 0%).

### Publication bias

Because of the small number of included studies (less than 10), we did not perform the Egger’s regression test and funnel plot observation due to a high risk of bias.

## Discussion

There have been robust efforts to decipher the molecular biomarkers of glioma and their prognostic significance as well as apply these findings to clinical practice, particularly in choosing appropriate candidates for initial chemotherapy [13, 30, 33–37]. *TERT* promoter mutation and MGMT methylation status are among the most important markers. MGMT promoter methylation is one of the few treatment-relevant markers, encoding an enzyme that removes mutagenic methylating lesions from the O^6^ guanine position. Methylation of the MGMT promoter leads to low expression of MGMT and inactivation of the repair protein, rendering tumor cells more sensitive to effects of alkylating agents [38]. Consequently, MGMT methylation is considered a favorable prognosis marker associated with longer survival outcomes [39].

Additionally, mutation in the *TERT* promoter has shown to have prognostic value across a range of tumors [4, 13, 33, 40–44]. Mutations in this promoter region maintain telomere length and tumor cell survival which plays a crucial role in cancer development [45]. Interestingly, high *TERT* activity occurs in 90% of human cancers [46], including gliomas (70%) [47].

Our study demonstrated that *TERT* promoter mutations showed contradicting effects in MGMT-meth and MGMT-unmeth gliomas. In MGMT-meth gliomas, *TERT* promoter mutation was correlated with a favorable survival outcome. In contrast, in MGMT-unmeth gliomas, *TERT* promoter mutation was regarded as an indicator of poor prognosis. From our results, the OS of gliomas can be further stratified into four distinct survival subgroups with ascending survival time as follow: *TERT*-mut/MGMT-unmeth << *TERT*-wt/MGMT-unmeth << *TERT*-wt/MGMT-meth << *TERT*-mut/MGMT-meth which is consistent with previous reports [16, 26]. This risk stratification will help clinicians better predict patient survival and tailor treatment decisions accordingly. However, the underlying mechanism on how MGMT promoter methylation modulates *TERT* promoter mutation has not been well elucidated. In one recent study, the *TERT*-mut/MGMT-unmeth GBM was associated with worse magnetic resonant imaging (MRI) characteristics such as low apparent diffusion coefficient values, obvious edema, obvious necrosis, unobvious non-contrast enhancing tumor, deep white matter invasion, and a high Ki-67 labeling rather than other groups [10]. On the other hand, it is interesting to note that *TERT* promoter mutation is an independent prognostic marker in other cancers (e.g., melanoma, thyroid cancer, urothelial carcinoma) and is not influenced by other mutations such as *RAS* or *BRAF* mutations [43, 44, 48–50]. In gliomas, the prognostic impact of *TERT* promoter mutation has been known to be modulated by *IDH* mutations [13]. Therefore, the principal concept of these modulations in glioma warrants further mechanistic investigation. In contrast to *TERT* promoter mutation, the prognostic impact of MGMT methylation was not dependent on other confounding factors including the status of *TERT* promoter mutation, emphasizing the important role of MGMT methylation as an independent prognostic marker in gliomas.

While the positive prognosis role of MGMT methylation in patients treated with TMZ has been observed in many studies [9, 36, 51–54], there were still conflicting results regarding the prognostic value of this genetic marker in GBM patients [34, 55]. It raises the question that there might be other factors affecting the responsiveness to TMZ besides MGMT methylation status. Our results led us to the observation that *TERT* promoter mutation was associated with the MGMT methylation benefit in GBM patients treated by TMZ whereas, in the *TERT-*wt group, MGMT methylation was not associated with improved OS in these patients. As a result, it is crucial to test for *TERT* promoter mutation and MGMT methylation in GBM patients who are eligible for TMZ chemotherapy.

The biological mechanism of interaction between *TERT* promoter mutation and MGMT methylation that may influence sensitivity to TMZ treatment of gliomas has not yet clearly defined. We believe that the efficacy of TMZ depends on both telomerase hyperactivity and muted MGMT gene expression. Based on our results, we assumed that MGMT promoter methylation might increase sensitivity to TMZ, mainly in the context of *TERT* promoter mutation. MGMT encodes an enzyme that removes alkylating lesions added by TMZ from the O6 guanine position. Methylation of MGMT promoter leads to low expression of MGMT and silence of repair protein, which makes tumor cells more sensitive to effects of TMZ [56]. Consequently, MGMT methylated status is considered a favorable prognostic marker associated with longer survival outcomes [8, 9, 57, 58]. Our immune system’s response to tumor may be in play as well. TMZ may improve tumor antigen presentation to T lymphocytes in a process known as cross-priming [59]. The facilitation of cell division by the *TERT* promoter mutation may lead cancerous cells to divide more quickly, divide, the more cell death and tumor lysis occur, which might increase releasing of tumor antigen. As a result, patients harboring *TERT* promoter mutation and MGMT methylation might show survival benefit with TMZ. Further investigation is required to understand clearly how these two genetic markers influence treatment response. In the unmethylated MGMT subgroup, TMZ’s cytotoxic alkylating effect is counteracted by the DNA repair enzyme. Other studies have also shown no significant survival benefit of TMZ chemotherapy in MGMT unmethylated patients [8, 9, 60].

Acknowledging minimal heterogeneity, we believe that our meta-analysis provides robust and useful directionality regarding the potential interaction between *TERT* and MGMT in glioma patients. However, we acknowledge that our meta-analysis is mainly based on retrospective studies which can lead to unavoidable selection biases. Moreover, our results were calculated from both individual and aggregate level data. While we attempted to minimize the differences in demographic and therapeutic data among the included studies by adjusting for various covariates, it should be noted that there might still be some discrepancies among different datasets such as molecular profiling of other genetic markers, tumor locations, and salvage therapies throughout the treatment of patients. It is of interest to perform subgroup analyses regarding effects of *TERT* promoter subtypes (C228T versus C250T) on patient OS. However, these data were only provided in two studies which is insufficient for further analysis.

## Conclusions

In summary, *TERT* promoter mutation should not be used as a single predictive factor in gliomas. Instead, it should be interpreted in combination with MGMT methylation status. In addition, *TERT* promoter mutation seems to be a useful biomarker in clinically evaluating sensitivity to TMZ for treatment of glioma patients who carry MGMT methylated status.

## Supplementary information

**Additional file 1.** Table 1. The PRISMA checklist

## Data Availability

Not applicable.

## Abbreviations

CI: Confidence interval
GBM: Glioblastoma
HR: Hazard ratio
IPD: Individual patient data
KMC: Kaplan Meier curve
OS: Overall survival
LGG: Lower-grade glioma
MGMT: O^*6*^*-methylguanine-DNA methyltransferase*
MGMT-meth: MGMT-methylated
MGMT-unmeth: MGMT-unmethylated
*NOS*: *Newcastle-Ottawa Scale*
TERT: Telomerase reverse transcriptase
TERT-mut: TERT-mutated
TERT-wt: TERT-wild-type
TMZ: Temozolomide
WHO: World Health Organization
